# Affective valence facilitates spatial detection on vertical axis: shorter time strengthens effect

**DOI:** 10.3389/fpsyg.2015.00277

**Published:** 2015-03-24

**Authors:** Jiushu Xie, Yanli Huang, Ruiming Wang, Wenjuan Liu

**Affiliations:** ^1^Center for Studies of Psychological Application, School of Psychology, South China Normal University, GuangzhouChina; ^2^Department of Educational Psychology, The Chinese University of Hong Kong, Hong KongChina

**Keywords:** embodied cognition, conceptual metaphor theory, grounded cognition, valence-space metaphor, affective word, spatial axis, interstimulus interval

## Abstract

Affective concepts can be described in terms of space, which is known as the valence-space metaphor. Previous studies have not investigated either the specifics of this metaphor on the transverse and vertical axes or the time course of this metaphoric association. With Chinese participants, we used a spatial cue task to study the valence-space metaphor on the transverse (left-and-right; Experiment 1A) and vertical (upper-and-lower; Experiment 1B) axes. After being shown an affective word and asked to keep it in mind, the participants were given a spatial target detection task. The results revealed that the metaphoric association was only found on the vertical axis. More specifically, keeping a positive word in mind facilitated the detection of the upper target, but no such effect was found in the detection of the lower target. Furthermore, in Experiment 2, we manipulated the duration of time (100, 500, and 1000 ms) between the offset of the affective word and the onset of the spatial target (i.e., interstimulus intervals, ISI), to test the dynamic time course of the valence-space metaphor on the vertical axis. The results showed that when ISI was 100 ms, keeping a positive word in mind facilitated the detection of the upper target and keeping a negative word in mind facilitated the detection of the lower target. However, when the ISI was 500 or 1000 ms, keeping a positive word in mind facilitated the detection of the upper target and no such effect was found in the detection of the lower target, indicating that ISI might be important in the valence-space metaphoric association. In sum, we found that the processing of affective valence activated the vertical spatial axis but not the transverse axis. Further, the association might be modulated by ISI, indicating that it may be related to attention allocation.

## Introduction

People frequently use space to understand emotion. When we say, “s/he is in low/high spirits," we are employing down or up to interpret sadness or happiness, respectively. Previous studies have found this valence-space metaphoric association along the vertical axis (up vs. down; Meier and Robinson, 2004; Schubert, 2005; Lakens, 2012; Gozli et al., 2013a). Moreover, other studies have also found a relationship between valence and dominant/non-dominant hands such that positive emotional valence relates to the dominant hand and negative emotional valence relates to the non-dominant hand (Casasanto, 2009; de la Vega et al., 2013). However, the valence-space metaphoric association along the transverse axis (left vs. right side) has remained unclear. More importantly, the dynamic change of this metaphoric association is still unknown. We aimed to test these questions in the experiments described below.

Concepts are the basis for human cognition. However, there are no concrete examples for abstract concepts. How then can we understand and represent abstract concepts? The idea of embodied cognition is that abstract concepts (e.g., affective concepts) could be grounded in sensorimotor systems (Niedenthal, 2007; Barsalou, 2008). Vermeulen et al. (2007) found that verifying properties from different modalities (i.e., visual, auditory, and affective systems) led to longer reaction times and higher error rates, known as switching costs, indicating that affective concepts were grounded in sensorimotor systems. Furthermore, verifying affective properties but not sensory properties of concepts resulted in an interference effect when participants kept the affective load (i.e., emotional faces) in mind (Vermeulen et al., 2014). Hence, with embodied cognition, processing affective concepts operates perceptual symbols in sensorimotor systems.

Like the embodied view, Lakoff and Johnson (1980) propose that people use metaphor to understand abstract concepts. With metaphor, people use source concepts (usually concrete concepts) to elaborate other target concepts (usually abstract concepts). Take affective words, for example. We often use space (concrete concepts) to elaborate affective valence (abstract concepts), such as saying, “you bring me up/down.” In this sentence, we understand “happy is up and sad is down” or “good is up and bad is down.” Hence, when we understand, and express positive and negative emotion, we also activate upper and lower spatial information, respectively.

Many studies have found this metaphoric association with spatial information in processing affective words along the vertical axis. Meier and Robinson (2004) found that discrimination for stimuli in the upper position was facilitated following positive words, whereas discrimination for lower stimuli was facilitated following negative words. Gozli et al. (2013a; Experiment 2) tested the effect of stimulus-onset asynchrony (SOA) on the valence-space metaphor. Priming stimuli (i.e., affective or non-affective words) appeared at the center of the screen for either a short or long SOA. Then a visual target letter “X” or “O” appeared above or below the priming stimuli. The participants identified the visual target regardless of whether the priming stimuli were affective or non-affective words. Identification was facilitated when upper spatial targets followed positive words or lower spatial targets followed negative words.

In addition to the valence-space metaphor along the vertical axis, the association between affective valence and dominant/non-dominant hand has also been tested in many studies. Casasanto (2009) proposes a body-specificity hypothesis to interpret the association between body and mind. This hypothesis holds that left- and right-handed people use different ways to represent information. Taking affective valence, for example, right-handers associate positive with right and negative with left, whereas left-handers associate positive with left and negative with right. In other words, people with different dominant hands think of affective valence differently (Casasanto, 2011).

The association between affective valence and dominant/non-dominant hand is limited but stable. Research by de la Vega et al. (2012) found that this association along the transverse axis emerged only when participants made valence judgments and explicit mapping between valence and hand (left or right hand). Subsequently, de la Vega et al. (2013) asked participants to cross their hands and use their left/right hand (Experiment 1) or the left/right key (Experiment 2) to respond to affective words. In this way, their right hands were at the left side and left hands were at the right side. Hand and side carried incongruent information. They found that participants’ responses were facilitated only when they used their right hands to respond to positive words and left hands to respond to negative words, no matter where the hand was. This finding indicates that the valence-space metaphor is related to hand along the transverse axis (de la Vega et al., 2013).

However, it is still unclear whether the valence-space metaphor is related to the left/right side, relative to the body. Previous studies only asked participants to respond to affective words using their hands, so they only tested the association between affective valence and response hand. Given this instruction, participants might indicate a mapping between affective valence and hand (which is one part of the body). Yet, the association between affective valence and left/right side (which is not a part of the body) has not been well investigated. Whether the valence-hand bodily association would extend its influence to space (left or right side) needs further research. Hence, in the current Experiment 1A, we adopted a spatial cueing task to test whether processing affective words activated spatial information along the transverse axis (left/right side). Meanwhile, we also tested whether processing affective words activated spatial information along the vertical axis (up/down; Experiment 1B) in order to replicate previous studies.

A further question concerns how affective words affect spatial processing in the priming paradigm. One potential explanation may be that the processing of affective words might engage spatial attention (Gozli et al., 2013b). Thus, an affective word could be treated as a special type of endogenous cue. Studies on typical endogenous cues found that reaction times to targets were a function of SOA. Frischen and Tipper (2004) found that valid gaze cue improved performance (cueing effect) when the SOA was 200 but not 1200 ms, indicating such attention shifts were rapid, automatic, and transient. Fischer et al. (2003) presented a smaller digit (e.g., 1 or 2) or a larger digit (e.g., 8 or 9) at the center of a screen with two boxes at the left and right side. They found larger or smaller digits shifted participants’ attention to the right or left side, respectively. This effect was found when the interval before targets was 400 and 500 ms, but was decayed when the interval became longer. In a Simon task, cue-induced spatial coding was found when the SOA was 100, 500, or 1000 ms, which supported that there was a short-duration coding period (at least 1 s) before decay (Danziger et al., 2001). Hence, the cueing effect for typical endogenous cues lasts for a short period.

However, if we treat affective words as a special type of endogenous cue, it is unknown whether the valence-space association would be changed under different durations between the offset of the affective word and the onset of the spatial target—hereinafter called interstimulus intervals (ISIs). If affective words in a valence-space metaphoric association had a similar function as typical endogenous cues, this association would be changed under different ISIs. If this is true, we could predict that this association would become weak or disappear when the ISI becomes longer. Testing the time course of valence-space metaphor could help us to better understand the function of affective words and the dynamic change of this metaphoric association. Thus, in Experiment 2, we manipulated the ISI between affective words and targets to test the time course of valence-space metaphor.

As mentioned above, in Experiment 1A, we tested whether processing affective words affects a spatial detection task along the transverse axis; that is, whether the valence-space metaphoric association exists along the transverse axis. If it exists, the likely pattern might be that processing positive words facilitates detection for right spatial targets and processing negative words facilitates detection for left spatial targets. In Experiment 1B, we aimed to replicate the results of previous studies indicating that processing affective words affects spatial detection tasks along the vertical axis: specifically, that processing positive words facilitates detection for upper spatial targets and processing negative words facilitates detection for lower spatial targets. The ISI in Experiments 1A and 1B was fixed at 750 ms. Experiment 2 tested the time course of the valence-space metaphoric association along the vertical axis by manipulating the ISI between affective words and spatial targets (100, 500, or 1000 ms). If affective words have similar functions as typical endogenous cues, this allocation would rapidly decay after processing affective words, and the valence-space metaphoric effect would be found with short ISIs (e.g., 100 ms), but not long ISIs (e.g., 1000 ms). In other words, the processing of affective words may facilitate participants’ performance along the vertical axis when the affective valence is metaphorically congruent with the vertical position, and this facilitation effect may decay rapidly.

## Experiment 1A

In this experiment, we tested whether the metaphoric congruency effect exists on the transverse (left-to-right) axis (i.e., whether processing affective words affects spatial target detection tasks on the transverse axis), using a procedure in which participants kept an affective word in mind and completed a spatial target detection task on the transverse axis.

### Method

#### Participants

Forty-five students (mean age = 20.33 years, SD = 1.73 years, 38 of them female) from South China Normal University, Guangzhou, China, participated in this experiment for a small monetary compensation. They were recruited randomly by posting an advertisement on a campus forum. All of them had normal or corrected-to-normal vision. They participated in this experiment voluntarily and could withdraw at any time. All participants provided written informed consent prior to the experiment. The study was approved by the ethics review board of South China Normal University.

#### Materials

We used the same 240 affective words as those used by Xie et al. (2014), which were selected from the Chinese Affective Words System (CAWS). Half of them were positive, while the other half were negative (Wang et al., 2008). Positive and negative words were matched for number of first character strokes, number of second character strokes, number of word strokes, and word occurrence frequency. E-Prime 2.0 (Psychological Software Tools, Inc., Sharpsburg, PA, USA) was used for material presentation and data recording.

#### Research Design and Procedure

This experiment adopted a 2 (valence of affective word: positive vs. negative) × 2 (location of spatial target: left vs. right) full within-subjects design.

**FIGURE 1:**
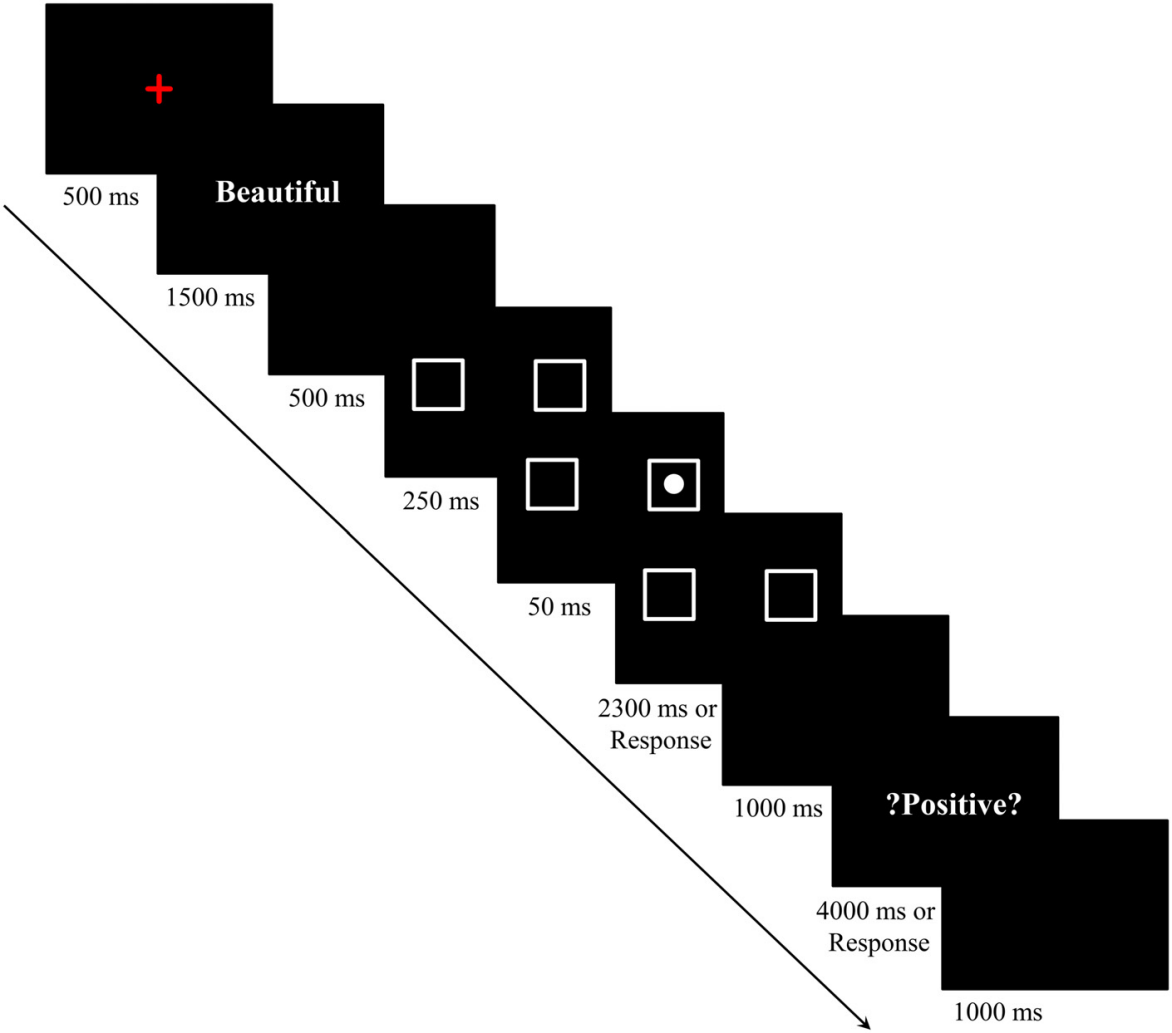
**Sequence of events in a trial of Experiment 1A (Chinese words have been translated into English)**.

The procedure was similar to the study of Ouellet et al. (2010; Experiment 1). The participants sat in a dimly lit soundproof cube. All materials were presented on a black background. To begin, a red cross was presented at the center of the screen for 500 ms, followed by a Chinese affective word (bold, 24-point SimSun Font) presented at the center of the screen for 1500 ms. The participants were instructed to memorize the word and that they would be tested at the end of each trial. After a 500-ms blank screen, two empty white squares (1.3 cm × 1.3 cm) were presented side by side, each appearing separately at the left and right of the screen for 250 ms. Then a white dot (diameter = 5 mm) appeared in one of these two squares for 50 ms. The squares remained on the screen for 2300 ms or until the participants made a response indicating the location of the white dot. Participants were asked to put their left index fingers on the “Z” key and right index fingers on the “M” key on the keyboard. They were asked to press the “Z” key to respond, if the dot appeared in the left square or press the “M” key to respond, if the dot appeared in the right square. After that, a blank screen was presented for 1000 ms. Then a question “? Positive?” or “?Negative?” was presented in Chinese for 4000 ms or until responses were given. The participants needed to judge whether this word (i.e., positive or negative) described the valence of the previously shown affective word correctly. Participants were asked to press the “Yes” key (“Z” or “M”) on the keyboard to respond if the question word described the valence of the previous word correctly or to press the “No” key (“M” or “Z”) to respond, if the question word described the valence of the previous word incorrectly. The allocation of “Yes” or “No” to “Z” or “M” was counterbalanced between participants. After a 1000-ms blank screen, the next trial was presented. Three short rest sessions were included in the experiment (see **Figure 1**).

Reaction times and accuracy data from the spatial detection task were dependent variables and were used for further analyses in all experiments reported here. We included the affective word memory test to ensure that participants memorized the affective words correctly in each trial.

### Results

Given that we used the same paradigm as that used in Ouellet et al. (2010; Experiment 1), we also adopted the same trimming and data analysis methods as those in Ouellet et al. (2010; Experiment 1). Two participants’ data were deleted due to their accuracies in the spatial target detection task or affective word memory task measuring lower than 75%. For the remaining participants, reaction times from the spatial detection task with erroneous trials (675 trials, 6.39%) in the detection and memory tasks were discarded. Then, correct trials with reaction times below 100 ms and above 650 ms (195 trials, 1.85%) were discarded as outliers. All remaining participants’ accuracy data from the spatial detection task were included in the analyses.

Reaction times and accuracy data from the spatial detection task were analyzed with two separate 2 (valence: positive vs. negative) × 2 (location: left vs. right) ANOVAs, using both participants (*F*_1_) and items (*F*_2_) as random factors. Valence was a within-subjects and between-items factor when participants and items were random factors, respectively. For studies on the valence-space metaphor, it is common to conduct two separate ANOVAs to include both participants and items as random factors in the analyses (e.g., de la Vega et al., 2012; Kong, 2013). Using the same data analyses as those used in previous studies makes it possible to compare the current results with those of previous studies.

No significant results were found in the accuracy data analyses (*p*s > 0.05). Specifically, the interaction between valence and target location was not significant, [*F*_1_(1,42) = 0.54, *p* = 0.468, _ηp^2^_ = 0.01; *F*_2_(1,238) = 0.48, *p* = 0.489, _ηp^2^_ < 0.01].

**FIGURE 2:**
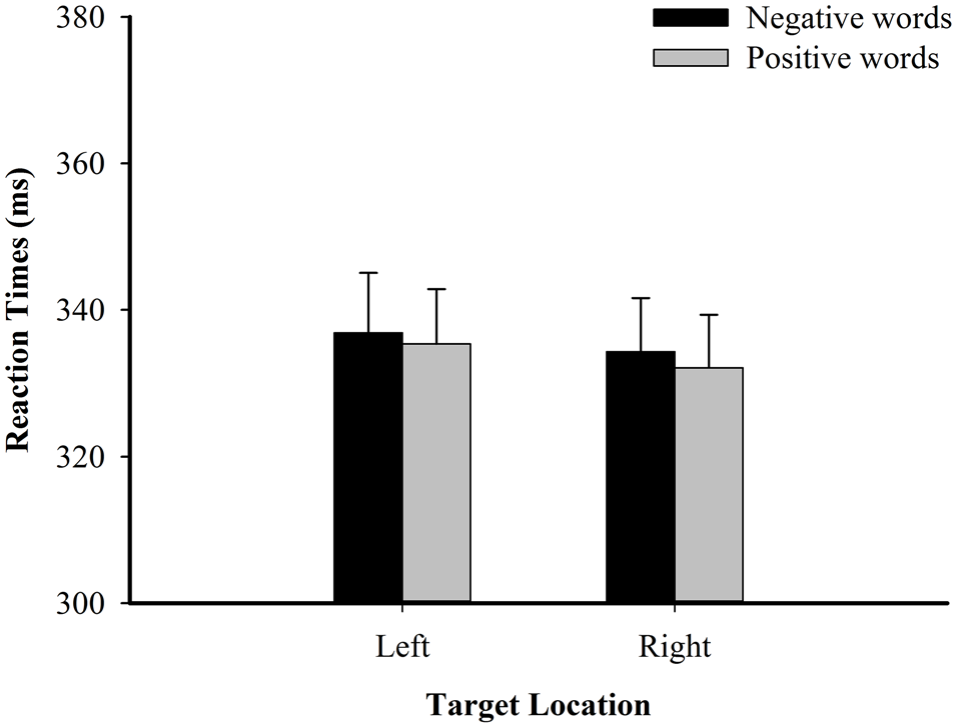
**Spatial target detection RT (in ms) in Experiment 1A**.

In the reaction times analyses, no significant results were found (*p*s > 0.05). Of particular note, the interaction between valence and target location was not significant, [*F*_1_(1,42) = 0.11, *p* = 0.748, _ηp^2^_ < 0.01; *F*_2_(1,238) = 0.60, *p* = 0.441, _ηp^2^_ < 0.01; see **Figure 2** and **Table 1**].

**Table 1:** Mean reaction times (ms) and correct responses (%) per condition in Experiment 1A.

	Affective words							
	Negative				Positive			
	Reaction times		Correct responses		Reaction times		Correct responses	
Target location	*M*	SE	*M*	SE	*M*	SE	*M*	SE
Left	336.87	8.19	99.40	0.23	335.34	7.51	99.24	0.24
Right	334.28	7.35	99.30	0.19	332.09	7.27	98.84	0.37

### Discussion

In Experiment 1A, we did not find any metaphoric congruency effects. Responses to the spatial target were not affected by affective words, indicating that participants may not use transverse space to understand affective words. In Experiment 1B, we intended to test whether participants use vertical (top-to-bottom) space to understand affective words. According to previous findings, metaphoric congruency effects have been reported with the vertical axis. We predicted that a metaphoric congruency effect would appear in Experiment 1B.

## Experiment 1B

In Experiment 1B, we try to replicate previous findings on metaphoric congruency effects using the same paradigm as Experiment 1A. The procedure was similar to Experiment 1A, except that the spatial target was presented on the vertical axis.

### Method

#### Participants

Forty-five students (mean age = 21.24 years, SD = 2.05 years, 35 of them female) from South China Normal University, Guangzhou, China, participated in this experiment voluntarily. None of them had participated in the previous experiment. They were recruited using the same methods as Experiment 1A. All participants provided written informed consent prior to the experiment and were paid after the experiment. The study was approved by the ethics review board of South China Normal University.

#### Materials

The same affective words were used as those in Experiment 1A.

#### Research Design and Procedure

This experiment also adopted a 2 (valence of affective word: positive vs. negative) × 2 (location of spatial target: bottom vs. top) full within-subjects design.

The procedure was similar to Experiment 1A, except for the following: (1) two empty white squares were presented at the bottom and top of the screen, (2) the white dot was presented in a bottom or top square, (3) participants used “Y” and “B” to respond to the spatial target detection task. When the dot was presented in the bottom or top square, participants pressed “Y” or “B” to make a response, respectively; (4) in the memory test task, participants pressed “Y” or “B” for a “Yes” or “No” response. The allocation of “Yes” or “No” to “Y” or “B” was counterbalanced between participants.

### Results

Two participants’ data were deleted due to their response accuracies in the spatial target detection task or affective word memory task measuring lower than 75%. The same trimming method was adopted as in Experiment 1A. First, erroneous trials (722 trials, 7.00%) in the detection or memory task were discarded. The remaining correct trials in the spatial detection task with reaction times below 100 and above 650 ms (200 trials, 1.94%) were also discarded as outliers.

Two separate 2 (valence of affective word: positive vs. negative) × 2 (location of spatial target: bottom vs. top) ANOVAs were employed to analyze reaction times and accuracy data from the spatial detection task. Both participants (*F*_1_) and items (*F*_2_) were considered random factors in the analyses.

In the accuracy data analyses, the main effect for location of spatial target was significant [*F*_1_(1,42) = 7.44, *p* = 0.009, _ηp^2^_ = 0.15; *F*_2_(1,238) = 14.00, *p* < 0.0005, _ηp^2^_ = 0.06]. Responses to upper targets were more accurate than lower targets. The interaction between valence of affective word and location of spatial target was not significant [*F*_1_(1,42) = 0.89, *p* = 0.351, _ηp^2^_ = 0.02; *F*_2_(1,238) = 0.53, *p* = 0.466, _ηp^2^_ < 0.01].

**FIGURE 3:**
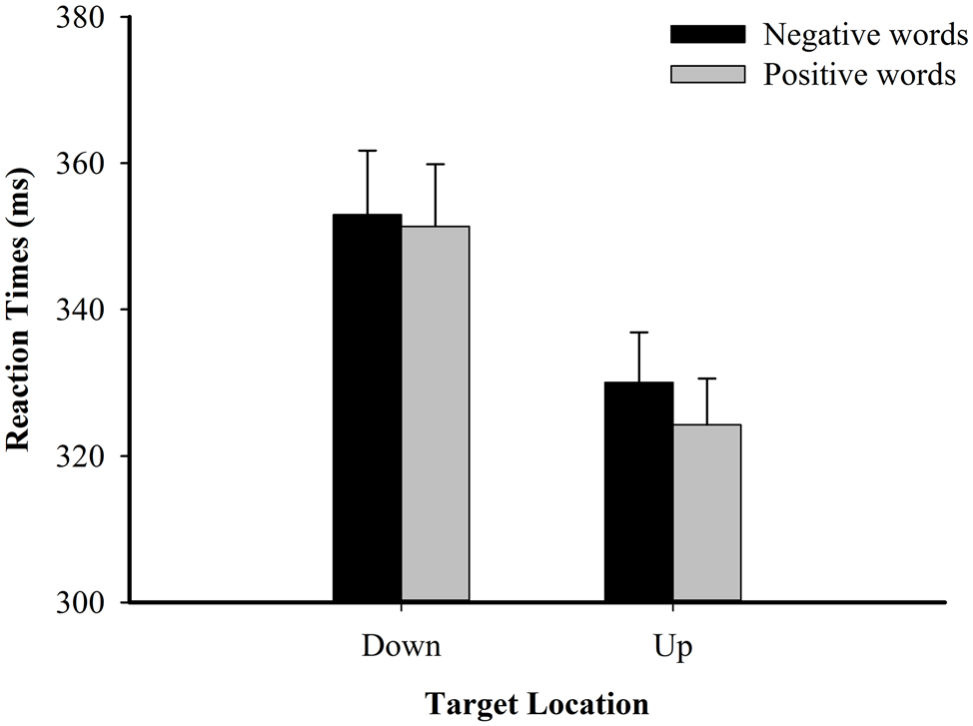
**Spatial target detection RT (in ms) as a function of affective valence in Experiment 1B**.

In the reaction times analyses, the main effect for valence of affective word was only significant by participant [*F*_1_(1,42) = 8.25, *p* = 0.006, _ηp^2^_ = 0.16; *F*_2_(1,238) = 2.35, *p* = 0.127, _ηp^2^_ = 0.01]. The main effect for location of spatial target was significant [*F*_1_(1,42) = 33.71, *p* < 0.0005, _ηp^2^_ = 0.45; *F*_2_(1,238) = 268.00, *p* < 0.0005, _ηp^2^_ = 0.53]. Importantly, the interaction between the valence of affective word and the location of spatial target was significant by item [*F*_1_(1,42) = 2.87, *p* = 0.097, _ηp^2^_ = 0.06; *F*_2_(1,238) = 4.29, *p* = 0.039, _ηp^2^_ = 0.02]. Further simple analyses found that when the spatial targets were presented at the top, positive words facilitated the participants’ responses [*F*_1_(1,42) = 9.62, *p* = 0.003, _ηp^2^_ = 0.19; *F*_2_(1,238) = 5.64, *p* = 0.018, _ηp^2^_ = 0.02], compared to negative words. No such effect was found when the spatial targets were presented at the bottom [*F*_1_(1,42) = 0.87, *p* = 0.357, _ηp^2^_ = 0.02; *F*_2_(1,238) = 0.02, *p* = 0.884, _ηp^2^_ < 0.01]. No further effects reached statistical significance (*p*s > 0.05; see **Figure 3**; **Table 2**).

**Table 2:** Mean reaction times (ms) and correct responses (%) per condition in Experiment 1B.

	Affective words							
	Negative				Positive			
	Reaction		Correct responses		Reaction times		Correct responses	
Target location	*M*	SE	*M*	SE	*M*	SE	*M*	SE
Down	352.96	8.78	99.19	0.22	351.37	8.51	99.30	0.24
Up	330.11	6.78	98.57	0.31	324.29	6.34	98.41	0.37

### Discussion

In Experiment 1B, we found a metaphoric congruency effect when the spatial target was presented at the top. Processing positive words facilitated participants’ responses to upper spatial targets, indicating that positive words may activate upper spatial information. However, we did not find any such congruency effect when the spatial target was presented at the bottom. In the experiment by Gozli et al. (2013b), processing positive words made participants’ saccade trajectories switch above fixation. Hence, our current findings are consistent with previous findings.

However, as it was not clear whether the metaphoric congruency effect on the vertical axis observed in Experiment 1B would be modulated by the ISI (i.e., the duration between the offset of the affective word and the onset of the spatial target), we intended to test this hypothesis in Experiment 2.

## Experiment 2

In Experiment 1B, we found a metaphoric congruency effect on the vertical axis. In the following experiment, we intended to test whether this effect would be modulated by the interval between the offset of the special endogenous cue (i.e., affective word) and the onset of the spatial target. Hence, we manipulated the ISI for 100, 500, or 1000 ms in the following experiment.

### Method

#### Participants

Fifty students (mean age = 21.40 years, SD = 1.80 years, 44 of them female) from South China Normal University, Guangzhou, China, participated in this experiment voluntarily. All of them were right-handed and had normal or corrected-to-normal vision. None of them had participated in previous experiments. They were recruited using the same methods as Experiment 1. All participants provided written informed consent prior to the experiment. The study was approved by the ethics review board of South China Normal University.

#### Materials

In order to ensure enough numbers of valid trials under each condition, we selected 288 affective two-character words from the CAWS (Wang et al., 2008). Words were matched for number of first character strokes (*M*_positive_ = 8.83, *M*_negative_ = 8.83), number of second character strokes (*M*_positive_ = 8.62, *M*_negative_ = 8.54), number of word strokes (*M*_positive_ = 17.44, *M*_negative_ = 17.38), and word occurrence frequency (*M*_positive_ = 68.26, *M*_negative_ = 62.56), all of which were statistically equivalent between positive and negative words (*p*s > 0.05).

#### Research Design and Procedure

This experiment adopted a 2 (valence of affective word: positive vs. negative) × 2 (location of spatial target: bottom vs. top) × 3 (ISI: 100, 500, or 1000 ms) full within-subjects design.

**FIGURE 4:**
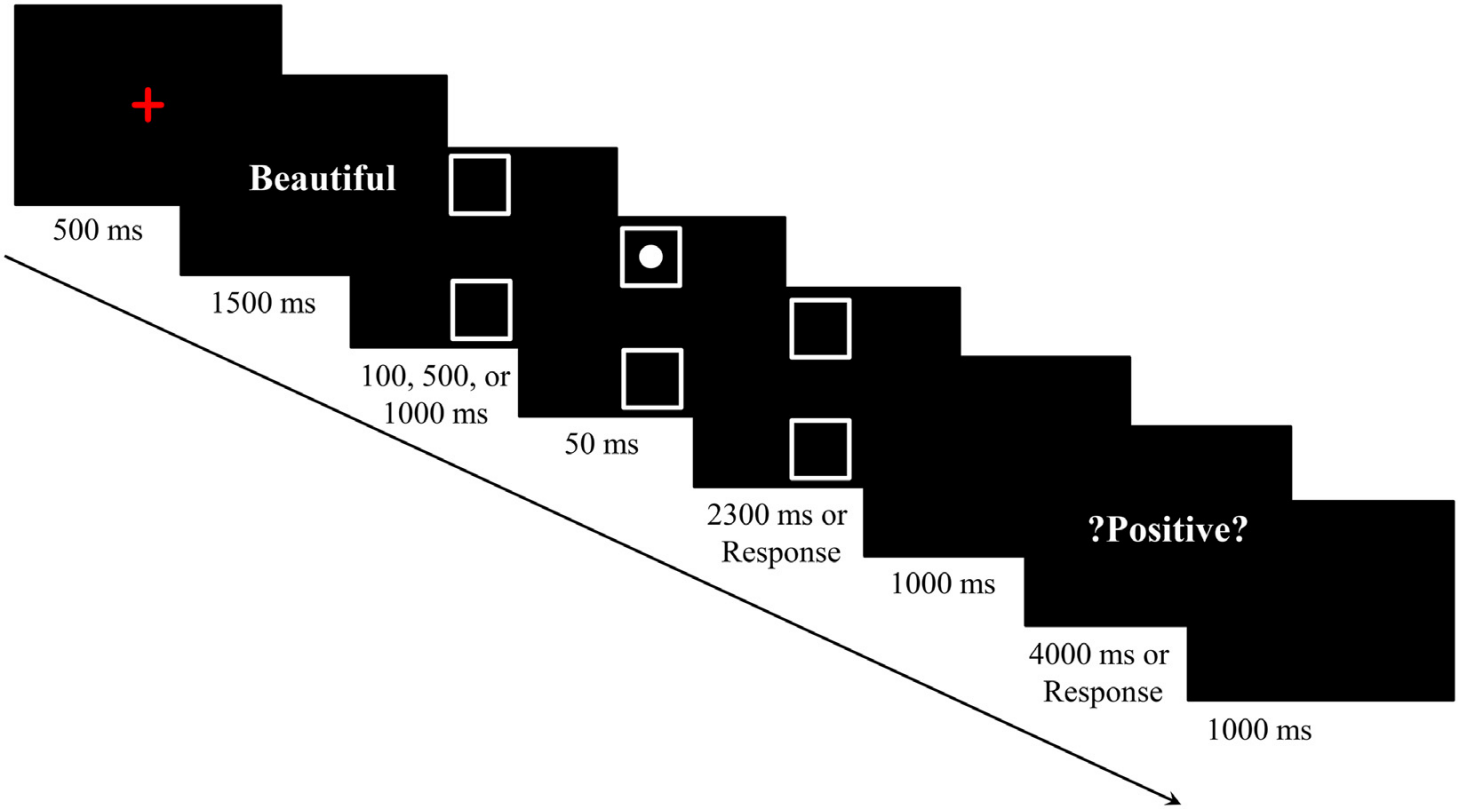
**Sequence of events in a trial of Experiment 2 (Chinese words have been translated into English)**.

The procedure was modified from Experiment 1B. We removed the blank screen after the affective word, and the duration of the empty boxes was 100, 500, or 1000 ms. In other words, we manipulated the duration between the offset of the affective word and the onset of the spatial target (i.e., the duration of the empty square boxes), which was the ISI in Experiment 2. Other aspects were the same as in those Experiment 1B (see **Figure 4**).

### Results

We used the same trimming method as that in Experiment 1. Data from two participants were removed because their accuracy was lower than 75% in the spatial target detection task or affective word memory task. First, erroneous trials (983 trials, 7.11%) in the detection or memory task were deleted. Correct trials in the spatial detection task with reaction times below 100 ms and above 650 ms (569 trials, 4.12%) were deleted as outliers.

Accuracy data and reaction times from the spatial detection task were submitted to two separate 2 (valence of affective word: positive vs. negative) × 2 (location of spatial target: bottom vs. top) × 3 (ISI: 100, 500, or 1000 ms) ANOVAs. Participants (*F*_1_) and items (*F*_2_) were both considered random factors in the analyses.

Accuracy analyses found the main effect for ISI was only significant by item [*F*_1_(2,94) = 3.29, *p* = 0.063, _ηp^2^_ = 0.07; *F*_2_(2,572) = 5.28, *p* = 0.006, _ηp^2^_ = 0.02]. The participants’ responses were most accurate when the ISI was 500 ms and least accurate when the ISI was 1000 ms. No other significant effects were found (*p*s > 0.05).

**FIGURE 5:**
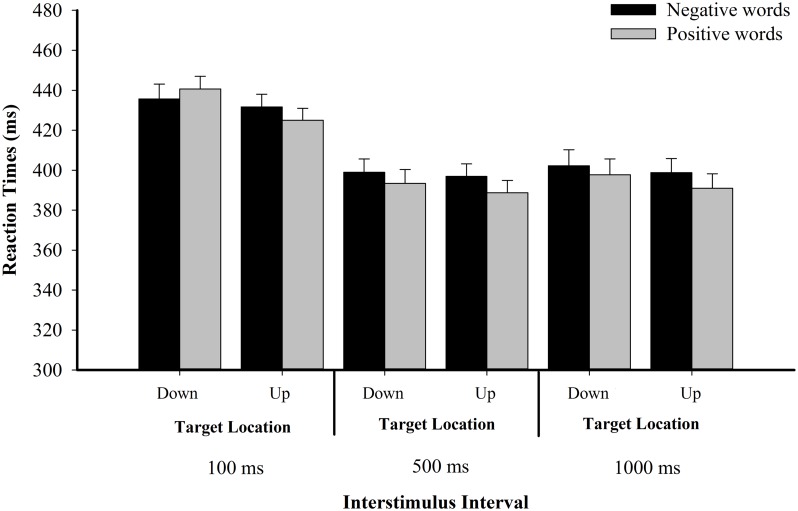
**Spatial target detection RT (in ms) as a function of affective valence and interstimulus interval (ISI) in Experiment 2**.

Reaction times analyses found the main effect for valence was only significant by participant [*F*_1_(1,47) = 13.08, *p* = 0.001, _ηp^2^_ = 0.22; *F*_2_(1,286) = 2.70, *p* = 0.101, _ηp^2^_ = 0.01]. The main effect for the location of spatial target was significant [*F*_1_(1,47) = 4.27, *p* = 0.044, _ηp^2^_ = 0.08; *F*_2_(1,286) = 8.90, *p* = 0.003, _ηp^2^_ = 0.03]. The main effect for ISI was significant [*F*_1_(2,94) = 114.957, *p* < 0.001, _ηp^2^_ = 0.71; *F*_2_(2,572) = 344.202, *p* < 0.001, _ηp^2^_ = 0.55]. The interaction between valence and location was significant [*F*_1_(1,47) = 4.15, *p* = 0.047, _ηp^2^_ = 0.08; *F*_2_(1,286) = 4.06, *p* = 0.045, _ηp^2^_ = 0.01]. Further simple effect analyses revealed that when the spatial targets were presented at the top, positive words facilitated the participants’ responses [*F*_1_(1,47) = 12.87, *p* = 0.001, _ηp^2^_ = 0.22; *F*_2_(1,286) = 7.16, *p* = 0.008, _ηp^2^_ = 0.02], compared to negative words. No such effect was found when spatial targets were presented at the bottom [*F*_1_(1,47) = 0.98, *p* = 0.327, _ηp^2^_ = 0.02; *F*_2_(1,286) = 0.03, *p* = 0.875, _ηp^2^_ < 0.01]. The three-way interaction between valence, ISI, and the location of spatial target was not significant [*F*_1_(2,94) = 1.71, *p* = 0.187, _ηp^2^_ = 0.04; *F*_2_(2,572) = 1.24, *p* = 0.289, _ηp^2^_ < 0.01]. No other significant effects were found (*p*s > 0.05; see **Figure 5**; **Table 3**).

**Table 3:** Mean reaction times (ms) and correct responses (%) per condition in Experiment 2.

		Affective words							
		Negative				Positive			
		Reaction times		Correct responses		Reaction times		Correct responses	
Interstimulus interval	Target location	*M*	SE	*M*	SE	*M*	SE	*M*	SE
100 ms	Down	435.69	7.44	98.35	0.76	440.68	6.34	98.70	0.50
100 ms	Up	431.71	6.35	98.70	0.61	425.00	5.99	98.79	0.37
500 ms	Down	399.00	6.66	99.31	0.26	393.42	6.92	99.13	0.28
500 ms	Up	396.89	6.30	98.61	0.55	388.75	6.17	99.05	0.31
1000 ms	Down	402.24	8.02	98.26	0.61	397.70	7.99	98.44	0.54
1000 ms	Up	398.86	6.99	98.26	0.62	390.96	7.25	97.83	0.73

We further conducted planned analyses to test whether interactions between valence and location were consistent under different ISIs. Results revealed that when the ISI was 100 ms, the interaction between valence and location was significant [*F*_1_(1,47) = 7.48, *p* = 0.009, _ηp^2^_ = 0.14; *F*_2_(1,286) = 7.08, *p* = 0.008, _ηp^2^_ = 0.02]. However, the interaction between valence and location was not significant when the ISI was 500 ms [*F*_1_(1,47) = 0.32, *p* = 0.572, _ηp^2^_ = 0.01; *F*_2_(1,286) = 0.45, *p* = 0.501, _ηp^2^_ < 0.01] and when the ISI was 1000 ms [*F*_1_(1,47) = 0.68, *p* = 0.415, _ηp^2^_ = 0.01; *F*_2_(1,286) = 0.54, *p* = 0.462, _ηp^2^_ < 0.01]. Simple effect analyses on the condition of 100-ms ISI found that when spatial targets were presented at the top of the screen, the facilitation effect of processing positive words on participants’ responses was significant by participant [*F*_1_(1,47) = 5.45, *p* = 0.024, _ηp^2^_ = 0.10; *F*_2_(1,286) = 2.60, *p* = 0.108, _ηp^2^_ = 0.01]. Such a facilitation effect of processing negative words on responses to the bottom spatial targets was also marginally significant [*F*_1_(1,47) = 2.87, *p* = 0.097, _ηp^2^_ = 0.06; *F*_2_(1,286) = 3.07, *p* = 0.081, _ηp^2^_ = 0.01]. However, we only found a facilitation effect of processing positive words with upper spatial targets when the ISI was 500 ms [*F*_1_(1,47) = 5.05, *p* = 0.029, _ηp^2^_ = 0.10; *F*_2_(1,286) = 3.28, *p* = 0.071, _ηp^2^_ = 0.01] and 1000 ms [*F*_1_(1,47) = 8.05, *p* = 0.007, _ηp^2^_ = 0.15; *F*_2_(1,286) = 4.45, *p* = 0.036, _ηp^2^_ = 0.02]. No significant effect was found with lower spatial targets when the ISI was 500 and 1000 ms (*p*s > 0.05).

### Discussion

In this experiment, we found that when the ISI was 100 ms, keeping a positive word in mind facilitated the detection of the upper target, and keeping a negative word in mind facilitated the detection of the lower target. However, when the ISI was 500 or 1000 ms, keeping a positive word in mind facilitated the detection of the upper target, but no such effect was found in the detection of the lower target. Thus, the valence-space metaphoric association might be modulated by ISI. Further, in Experiment 1B, we also found positive words facilitated participants’ responses for upper spatial targets, relative to negative words, when ISI was 750. This finding is the same as the finding in Experiment 2 when the ISI was 500 or 1000 ms. Hence, we replicated the finding from Experiment 1B successfully.

## General Discussion

The present studies tested the metaphoric association along the transverse and vertical axes and further investigated dynamic variations of this association under different intervals between affective words and spatial targets. Experiment 1A did not reveal that processing affective words affected spatial detection along the transverse axis. However, Experiment 1B demonstrated that processing affective words did affect spatial detection along the vertical axis. Experiment 2 replicated the findings from Experiment 1B and further found that the interaction between valence and location along the vertical axis might be modulated by ISI. Together, results indicate that both axis and ISI might be important in the metaphoric association.

Experiment 1A did not find a valence-space metaphoric association along the transverse axis. However, the body-specificity hypothesis holds that right-handed people associate right with positive and left with negative, whereas left-handed people show a reversed pattern (Casasanto, 2009). This association even emerges in children and cannot be explained by language or cultural conventions (Casasanto and Henetz, 2012). The absence of metaphoric association in Experiment 1A does not contradict the body-specificity hypothesis. This hypothesis posits a natural association between valence (e.g., positive vs. negative) and dominant/non-dominant hand (i.e., body). However, Experiment 1A tested the relationship between affective valence (e.g., positive vs. negative) and left/right space (which is not a part of body, i.e., non-body). Hence, the body-specificity hypothesis posits bodily association, whereas our Experiment 1A tested non-bodily association; this may explain the difference between the prediction of the body-specificity hypothesis and our results.

Although we did not control for handedness in Experiments 1A,B, the absence of metaphoric association in Experiment 1A could not be attributed to handedness. First, all participants were Chinese. The percentage of left-handed people is only 0.23% among Chinese (Li, 1983). Considering that we recruited participants randomly for Experiments 1A,B, the probability of recruiting left-handed participants was very low. Second, in Experiment 2, we only recruited right-handed participants and found the same results as those in Experiment 1B. Hence, we believe that the results would remain unchanged if we controlled for handedness.

We found a metaphoric association along the vertical axis in Experiment 1B, when ISI was 750 ms. This metaphoric congruency effect was only found when spatial targets were presented at the upper location. Processing positive words facilitated the detection of upper spatial targets compared to negative words. We did not find that processing negative words facilitated the detection of lower spatial targets, in contrast to the results of Meier and Robinson (2004; Experiment 2). In their experiment, participants verbally evaluated affective valence followed by discriminating a non-valenced target (i.e., q or p) that appeared at the bottom or top of the screen. They found that responding to positive and negative words facilitated the discrimination of upper and lower targets, respectively. They interpreted this symmetric facilitation effect as a Stroop-like effect. The Stroop-like effect predicts that upper targets following positive words and lower targets following negative words will be processed faster than upper targets following negative words and lower targets following positive words. This prediction was not fully supported by our results in Experiments 1B.

On the contrary, Schubert (2005; Experiment 5B) asked participants to judge the affective valence of group names that appeared at the top or bottom of the screen. They only found that positive group names were evaluated faster when they were presented at the top of the screen compared to negative group names. No such effect was found when they were presented at the bottom of the screen. Hence, whether the metaphoric congruency effect is symmetric is debatable. Further studies are needed to test this question. However, this finding is compatible with our results in Experiment 1B.

The asymmetric metaphoric congruency effect observed in Experiment 1B may be interpreted by the polarity correspondence (Lakens, 2012). Proctor and Cho (2006) propose that the polarity correspondence could be used for interpreting mapping effects in binary choice reaction tasks. People code stimulus and response alternatives as positive (+polar) and negative (-polar) polar along several dimensions, which is a basic aspect of human information processing. People’s responses are facilitated to a greater extent when the polarities correspond rather than when they do not. Lakens (2012) extended the polarity correspondence into the metaphoric congruency effect. Lakens (2012) further manipulated the polarity frequency (75% +polar and 25% -polar, or vice versa) and found that the interaction between words and vertical position was removed in the 75% -polar condition, indicating that the metaphoric congruency effect could be explained by polarity correspondence.

In Experiment 1B, we only found a metaphoric congruency effect when spatial targets appeared at the top of the screen. This result is consistent with polarity correspondence, which holds that processing +polar (up) is faster than –polar (down). Only +polar spatial responses receive a polarity benefit but not –polar spatial responses (Lynott and Coventry, 2014). In the current experiment, positive words and upper spatial targets could be treated as +polar and negative words and lower spatial targets could be treated as -polar. Hence, only when spatial targets appear on the top of the screen (+polar), are responses facilitated by positive words (+polar), which is supported by the results of Experiment 1B.

In Experiment 1, we replicated previous findings and found that processing affective words facilitated spatial target detection along the vertical axis. However, we did not find this effect along the transverse axis. One potential explanation for this may be that, in Chinese culture, people treat the left side as more positive than the right side. However, the body-specificity hypothesis holds that the right side is more positive than the left side for right-handed people (Casasanto, 2009). This conflation between Chinese culture and the body-specificity hypothesis may eliminate the mapping between valence and left/right side. Further studies are needed to test this hypothesis.

In Experiment 2, we intended to test the time course of the metaphoric association. The results found that when ISI was 100 ms, processing upper targets was faster, following positive words compared to negative words. Meanwhile, this effect was also marginally significant for the processing of lower targets. On the contrary, when ISI was 500 or 1000 ms, we only found the facilitation effect of positive words on upper targets. The facilitation effect of negative words on lower targets disappeared. It seems that the metaphoric association was stronger with ISI of 100 ms. The symmetric facilitation effect of affective words on targets (i.e., positive words facilitated the processing of upper targets and negative words facilitated the processing of lower targets) only emerged at 100-ms ISI. This finding suggests that ISI is important in metaphoric association.

This metaphoric association observed in Experiments 1B and 2 is most likely based on attention allocation. Xie et al. (2014) adopted a similar paradigm using a 750-ms ISI and found that when spatial targets appeared at the top and bottom of the screen, a larger P200 amplitude was found after positive and negative words, respectively. This P200 may be related to attention allocation. Zhang et al. (2013) tested the neural mechanisms underlying spatial target discrimination after spatial cue words using a random ISI from 400 to 500 ms. Spatial cue words denoted objects that typically appeared in upper or lower space. The spatial targets were “p” or “q” that appeared at the top or bottom of the screen. Although the cue words did not predict discrimination of spatial targets, the authors still found enhanced N1 amplitudes for congruent conditions (e.g., “eagle” followed by upper spatial targets) compared to incongruent conditions (e.g., “eagle” followed by lower spatial targets). These findings indicated that the effect of processing of words on the subsequent spatial task might be based on spatial attention.

If the attention allocation interpretation is indeed a basis for metaphoric association in Experiments 1B and 2, this attention allocation may be different from attention allocation that is endogenously oriented by central cues. In Experiments 1B and 2, the central cues were affective words that did not contain valid spatial information for the subsequent spatial targets. In this view, the affective words were invalid cues. However, conceptual metaphor theory holds that affective valence is based on space: positive is based on upper space, whereas negative is based on lower space (Lakoff and Johnson, 1980). Vermeulen (2010) found that positive emotion decreased the attentional blink (the negative effect of identification of the first target on identification of the second target), whereas negative emotion increased it, implying that attention was distinctively affected by current affective states. Hence, affective words may be treated as special endogenous cues that may have typical cueing effects. The typical endogenous cueing effect was modulated by SOA. Funes et al. (2005) adopted color as a central symbolic cue in a spatial Stroop task. They found that the facilitation effect was maximal at an 850-ms SOA following central cues. The facilitation effect of typical endogenous cues would disappear after a long SOA. This may explain why interactions between the valence of affective word and the location of spatial target were different under short ISI (100 ms) versus long ISI (500 or 1000 ms) in Experiment 2. In addition, more studies are needed to test the difference between typical endogenous cues and special central cues (i.e., words that have metaphoric association with spatial information) in cueing tasks.

The present studies found that axis might be important in the valence-space metaphoric association. The role of axis in other metaphors has also been debated. Boroditsky et al. (2011) found that Chinese speakers prefer to think about time vertically and horizontally compared to English speakers who prefer to think about time horizontally. However, Tse and Altarriba (2008) revealed that both Chinese–English bilinguals and English monolinguals think about time vertically. From this debate, we could hypothesize that processing abstract concepts (e.g., emotion or time) mainly relies on the vertical spatial axis. The processing of abstract concepts in relation to the transverse spatial axis still needs further study. More studies on the development of metaphor may shed light on this question.

In Experiment 1, we used a between-subjects design to test the valence-space metaphor along the transverse and vertical axes because it is difficult for participants to respond using four keys simultaneously in one study. Follow-up studies may modify the current paradigm and adopt a non-hand-operated response in the experiment to test the three-way interaction between valence of affective word, location of spatial target, and axis directly within one experiment. Second, we did not test the valence-space metaphoric association along the transverse axis under different ISIs, because this association did not emerge under a moderate ISI in Experiment 1A (i.e., 750 ms). However, in Experiment 1B, this association emerged along the vertical axis at the same ISI. Further studies may test the time course of valence-space metaphoric association along the transverse axis. Third, in Experiment 2, we did not include a longer ISI (e.g., 2000 ms). The three-way interaction between valence of affective word, location of spatial target, and axis was not significant. Follow-up studies may consider replicating the current study including longer ISIs.

In sum, our studies found that axis might be important in the valence-space metaphor. The interaction between valence of affective word and location of spatial target only emerged along the vertical axis. Further, ISI might also be important in the valence-space metaphor. When ISI was 100 ms, positive words facilitated the detection of the upper targets, compared to negative words. Meanwhile, negative words facilitated the detection of the lower targets, compared to positive words. However, when the ISI was 500 or 1000 ms, results only revealed that positive words facilitated the detection of the upper targets, compared to negative words. No such effect was found in the detection of the lower targets. The current study thus provides evidence for the spatial specificity and time course of valence-space metaphors.

## Author Contributions

RW developed the study concept. JX and RW designed experiments. JX and YH ran data-collection procedures of Experiments 1A,B. JX and WL ran data-collection procedure of Experiment 2. JX and RW analyzed and interpreted the data. JX drafted the manuscript. RW and YH provided critical revisions for the manuscript.

## Conflict of Interest Statement

The authors declare that the research was conducted in the absence of any commercial or financial relationships that could be construed as a potential conflict of interest.
